# The Underestimated Role of Environmental Factors in the Prevention of Invasive Fungal Disease: Experience From a European Childhood Cancer Centre

**DOI:** 10.1111/myc.70204

**Published:** 2026-06-29

**Authors:** Stefano Malvestiti, Felicia Andresen, Markus Hufnagel, Carsten Speckmann, Brigitte Strahm, Tobias Feuchtinger, Alexander Puzik

**Affiliations:** ^1^ Department of Pediatric Hematology, Oncology and Stem Cell Transplantation, Children's Hospital Faculty of Medicine, Medical Center—University of Freiburg Freiburg Germany; ^2^ Division of Pediatric Hematology/Oncology, Dana‐Farber/Boston Children's Cancer and Blood Disorders Center, Department of Pediatrics Harvard Medical School Boston Massachusetts USA; ^3^ Department of General Pediatrics, Adolescent Medicine and Neonatology, Division of Pediatric Rheumatology and Clinical Infectious Disease Faculty of Medicine, Medical Center—University of Freiburg Freiburg Germany; ^4^ Center for Chronic Immunodeficiency (CCI), Institute for Immunodeficiency Medical Center—University of Freiburg, Faculty of Medicine, Albert‐Ludwigs‐University of Freiburg Freiburg Germany

**Keywords:** environmental prophylaxis, invasive fungal disease, paediatric oncology, pharmacological prophylaxis, stem cell transplantation

## Abstract

**Background:**

Immunocompromised children with hematologic malignancies or undergoing allogeneic haematopoietic stem cell transplantation (HSCT) are at high risk for invasive fungal diseases (IFDs). Reported incidence varies considerably due to heterogeneous diagnostic criteria, antifungal strategies and environmental conditions. Environmental preventive measures, although highly relevant, remain underrecognised determinants of IFD incidence.

**Methods:**

This retrospective, single‐centre trial included paediatric cancer or transplant patients at high risk for IFD treated before (Cohort 1) and after (Cohort 2) relocation of a paediatric cancer centre from a 1990s building to a state‐of‐the‐art facility with improved environmental protection standards (observation periods: 56 and 12 months, respectively). Antifungal prophylaxis continuously followed local standards. IFD was diagnosed according to 2019 EORTC/MSGERC criteria. Primary endpoint was IFD incidence; secondary endpoints included prophylaxis use, IFD management and mortality.

**Results:**

This study included 186 patients (Cohort 1: *n* = 140; Cohort 2: *n* = 46). Baseline characteristics were comparable between both cohorts. Adherence to prophylaxis standards exceeded 98%, with liposomal amphotericin B being the most common agent. In Cohort 1, 25 possible, probable, or proven IFD occurred, mainly pulmonary aspergillosis, whereas no cases were observed following implementation of environmental measures in Cohort 2 (17.9% vs. 0%, *p* = 0.002). Most IFD cases occurred in HSCT recipients. IFD was associated with increased mortality (*p* < 0.0001).

**Conclusions:**

In this contemporary paediatric cancer and transplant setting, environmental protective measures were associated with a marked reduction in IFD incidence, complementing consistent pharmacologic prophylaxis. These findings underscore environmental protection as essential for IFD prevention in high‐risk paediatric populations.

## 1 Introduction

Immunodeficient paediatric patients are at substantial risk of developing invasive fungal disease (IFD), which is associated with considerable morbidity and mortality. This risk is particularly high among patients with haematologic malignancies receiving intensive chemotherapy regimens, those undergoing allogeneic haematopoietic stem cell transplantation (HSCT) and children with immunodeficiencies [1, 2, 3].

Determining the real‐world incidence of IFD in paediatric oncology is challenging and reported rates vary widely. Firstly, diagnostic criteria are heterogeneous and IFD definitions across institutions and studies are applied inconsistently [2]. For instance, Rosen et al. documented a 4.9% incidence in over 1.000 children with cancer from a single US centre. However, the IFD cases also included patients presenting with mucosal fungal infections and the study excluded transplant patients [4]. Conversely, Kaya et al. reported a 13.6% incidence in Turkish children with leukaemia receiving fluconazole prophylaxis, applying diagnostic criteria of the European Organization for Research and Treatment of Cancer (EORTC) [5]. Beyond differences in IFD diagnosis, variable treatments, prophylaxis strategies and partly empirical choice of antifungal agents may contribute to the observed heterogeneity in incidence estimates [6]. Indeed, divergent antifungal prophylaxis practices between the Berlin‐Frankfurt‐Münster group (BFM) and the Children's Oncology Group (COG) have been considered responsible for differing IFD rates in comparable paediatric AML cohorts [7, 8, 9]. Similar discrepancies are evident in other international comparative studies, where the use of prophylactic antifungal agents varies substantially even within comparable risk groups [1, 10].

To harmonise IFD classification, the European Organization for Research and Treatment of Cancer and Mycoses Study Group Education and Research Consortium (EORTC/MSGERC) developed consensus definitions that standardise criteria for classifying IFD as proven, probable, or possible based on host factors, clinical features and mycological evidence [11]. In brief, proven IFD is confirmed when definitive evidence of fungal growth is obtained from a normally sterile site with evidence of tissue damage. Probable IFD requires the co‐occurrence of at least one host risk factor, at least one IFD‐typical clinical feature and one non‐cultural mycological evidence (e.g., galactomannan test, polymerase chain reaction [PCR], microscopical detection). Possible IFD is diagnosed when host and clinical criteria are met but mycological evidence is absent. These definitions serve as the current reference standard and facilitate comparability across studies.

Parallel efforts have been undertaken to standardise antifungal prophylaxis strategies. Recommendations from the Australasian Antifungal Guidelines Steering Committee (AAGSC), the European Society of Clinical Microbiology and Infectious Diseases (ESCMID) and other expert panels broadly concur on the indications for prophylaxis and the preferential use of mould‐active agents [6, 12, 13].

Alongside antifungal prophylaxis, environmental preventive measures represent the second major pillar of IFD prevention, aimed at minimising patient exposure to exogenous fungal spores—particularly airborne *Aspergillus* species. However, the available evidence stems primarily from retrospective analyses or outdated prospective trials conducted under hygienic and prophylactic standards that no longer reflect current practice [14, 15, 16, 17]. The most recent study evaluating protective environmental measures was published in 2013. A total of 128 cancer patients treated in well‐sealed rooms with high‐efficacy particulate air (HEPA)‐filters and positive air pressure were compared with a historic cohort (*n* = 124) lacking these measures. While a significant reduction in fungal infections was observed (4% vs. 12%), results were biased by lower rates of antimycotic prophylaxis in the historic cohort and the absence of risk‐group stratification [17]. Consequently, a rigorous evaluation of the impact of environmental preventive interventions on IFD incidence is warranted, particularly within contemporary clinical settings that accurately represent the state‐of‐the‐art in pharmacological prophylaxis.

Therefore, the aim of this study is threefold: (1) to determine the incidence of IFD using the current EORTC/MSGERC criteria in a large European paediatric cancer centre; (2) to evaluate local antifungal prophylaxis practices in the context of national and international recommendations and (3) to assess the impact of changing environmental preventive measures on IFD incidence in a modern, high‐standard medical environment. The latter was made possible by the planned relocation of the University of Freiburg Medical Center children's hospital from a 1990s‐era building to a modern building with state‐of‐the‐art facilities, which uniquely allowed for direct comparison of two cohorts in their environmental setting in the absence of other relevant bias.

## 2 Methods

### 2.1 Study Design

This retrospective, single‐centre trial included patients at high risk for developing IFD who were treated at the Department of Pediatric Hematology and Oncology of the Medical Center of the University of Freiburg in two different time periods (Cohort 1: between 1 January 2016 and 30 August 2020, and Cohort 2: between 1 October 2024 and 30 September 2025). Between 1 September 2020 and 30 September 2024, a new building for the children's university hospital was constructed. Patients treated in this time interval were intentionally excluded from the study, as extensive construction work for the new children's university hospital building in proximity as well as renovation and demolition work in the old building could have led to an artificial increase in fungal spore concentrations. Renovation work is a known risk factor for IFD [18]. One of the included patients was also part of another clinical trial (DRKS00024719) that investigated the role of surgical intervention in the treatment of pulmonary aspergillosis [19].

Patients received antifungal prophylaxis according to local standards which were adapted to international guidelines [12]. Standards remained unchanged between both cohorts, with the exception of fluconazole discontinuation in non‐high‐risk transplant patients (for details see ‘Local standards of procedure (SOP) for prophylaxis of invasive fungal disease’ in Supporting Information and Table S7).

According to the European Conference on Infections in Leukaemia (ECIL)‐8 recommendations, patients at high risk for IFD were defined as individuals undergoing HSCT or affected by one of the following conditions: acute myeloid leukaemia (AML), high‐risk acute lymphoblastic leukaemia (ALL), relapsed or refractory (r/r) ALL/AML/lymphoma, juvenile myelomonocytic leukaemia (JMML), chronic granulomatous disease (CGD) or persistent severe neutropenia (absolute neutrophil count [ANC] < 500/μL for > 10 days), particularly including cases of myelodysplastic syndrome (MDS), refractory cytopenia of childhood (RCC), or severe aplastic anaemia (SAA) [6]. Eligible patients were identified using the department's International Classification of Diseases (ICD)‐10 diagnosis registry and the department's ICD‐10 based database of presumed fungal infection used for all patients receiving antifungal prophylaxis. Final inclusion was determined by electronic cross‐referencing of ICD‐10 diagnosis codes and antifungal prescription records. Only high‐risk patients with cancer or undergoing HSCT routinely receive antifungal prophylaxis according to the local SOP. Annually, at least 30 stem cell transplants are performed in our institution, and approximately 100 patients are diagnosed with a new or relapsed oncologic disease, including 25%–30% with acute leukaemia, among whom 15%–20% exhibit high‐risk features. We therefore estimated that at least 180 patients would be eligible for inclusion in this study. Patients who were diagnosed and treated for IFD before referral or admission to the Department of Pediatric Hematology and Oncology of the Medical Center of the University of Freiburg were excluded from this study.

IFD were classified according to the 2019 revised EORTC/MSGERC criteria (Table S1) [11, 20]. Residual IFD was defined as the presence of microbiological, serological, or imaging evidence of IFD at the last follow‐up.

Primary endpoint was the incidence of IFD according to EORTC/MSG criteria in the two cohorts. Secondary endpoints included antifungal prophylaxis use, IFD management and mortality rate.

### 2.2 Environmental Conditions of the New Children's Hospital

To compare the environmental conditions of the new and old building please see Table S2. Construction projects of the new children's hospital were designed in accordance with the 2010 recommendations of the German Commission for Infection Prevention and Hygiene in Healthcare and Nursing (KRINKO) to ensure compliance with nationally mandated hygienic standards for the treatment of immunosuppressed individuals [21]. The new children's hospital fully meets all KRINKO requirements. Room allocation follows the three‐tier risk classification for immunosuppressed patients defined by KRINKO in 2010 and reaffirmed in 2022 [21, 22]. Wards hosting immunocompromised patients are located in a separate unit, physically distant from the passageways to other departments. Patients classified as KRINKO risk group 1 and 2 are accommodated in double rooms with shared en‐suite sanitary facilities [22]. Individuals undergoing allogeneic HSCT (KRINKO risk group 3) are placed in single rooms with sealed windows, private adjacent sanitary facilities, positive room pressure and a negative‐pressure anteroom (with two doors) separating the patient room from the central aisle of the ward. An additional two‐door physical barrier isolates the aisle of the HSCT area from the remainder of the oncology ward. All rooms, anterooms and the aisle within the HSCT area are equipped with high‐efficiency particulate air (HEPA)‐filters (filter class H13). Air flow is directed from the patients' vicinity toward the exhaust vents. The entire area is subject to regular monitoring, including annual filter maintenance and air‐pressure surveillance. Daily cleaning of sanitary facilities and surfaces is ensured.

### 2.3 Data Collection

Clinical, imaging, laboratory and microbiological data of all patients were extracted from the digital medical data and information system, including age, gender, underlying type of cancer, type of chemotherapy or conditioning regimen, duration of neutropenia, time to engraftment and survival. In addition, prophylactic, pre‐emptive or targeted antifungal treatment including agents and duration of treatment were recorded.

### 2.4 Statistical Analysis

Data are reported as frequency (n), percentage (%), median, mean, standard deviation (SD) and interquartile range (IQR). For descriptive statistics, Chi‐square‐test and unpaired *t*‐test were performed, whenever required. A *p* value of < 0.05 was considered statistically significant. Odds ratio (OR) with 95% CI was used to quantify the association between investigated variables and the occurrence of IFD, or mortality. The statistical analysis was performed using Graphpad Prism (Version 10.1.2, GraphPad Software, Boston, Massachusetts USA).

### 2.5 Ethics Statement

The study was conducted in accordance with the Declaration of Helsinki and approved by the Institutional Review Board of Albert‐Ludwigs‐University Freiburg (IRB‐Number: 26‐1198‐S1‐retro). Written informed consent for data analysis and publication was obtained from the patients and/or their legal guardians at the time of cancer diagnosis or transplant preparation. All reasonable measures were taken to protect patients' anonymity.

## 3 Results

### 3.1 General Characteristics

**FIGURE 1:**
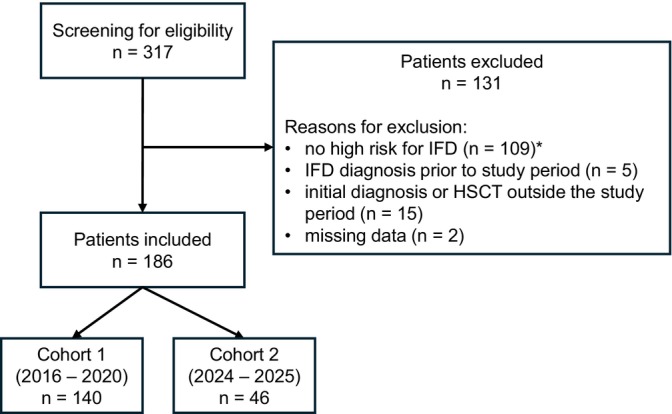
Flowchart of patient selection. *The majority of the excluded patients were initially identified as ALL; following further scrutiny, they did not display any high‐risk features and were therefore finally not included in this study. ALL, acute lymphoblastic leukaemia; HSCT, haematopoietic stem cell transplantation; IFD, invasive fungal disease.

Our ICD‐10 code‐based search identified a total of 317 patients: 242 belonged to Cohort 1 and 75 to Cohort 2 (see Figure 1 and study design). Among these, only 140 and 46 patients, respectively, met all inclusion criteria. Patient characteristics of the final 186 patients are summarised in Table 1. Cohorts 1 and 2 were comparable in age, gender, underlying disease and HSCT rate. The only statistically significant difference was observed in the prevalence of inherited bone marrow failure (BMF) syndromes (6.4% in Cohort 1 and 17.4% in Cohort 2; *p* < 0.05).

**TABLE 1:** Patient characteristics.

	Cohort 1	Cohort 2
Study population (*n*)	140	46
Sex (*n*, %)		
Female	59 (42.1%)	20 (43.5%)
Male	81 (57.9%)	26 (56.5%)
Age (median; IQR, years)	5.0 (1.3–12.1)	5.5 (1.4–9.0)
Underlying condition (*n*, %)		
Malignancy		
ALL HR	19 (13.6%)	6 (13.0%)
AML	20 (14.3%)	5 (10.9%)
r/r Leukaemia	16 (11.4%)	5 (10.9%)
JMML	4 (2.9%)	0 (0.0%)
r/r Lymphoma	4 (2.9%)	1 (2.2%)
MDS‐EB	3 (2.1%)	2 (4.3%)
Bone marrow failure (*n*, %)		
RCC	20 (14.3%)	3 (6.5%)
SAA	3 (2.1%)	2 (4.3%)
Inherited BMF^a^	9 (6.4%)	8 (17.4%)*
Immunodeficiencies		
CGD	7 (5.0%)	4 (8.7%)
CID^b^	4 (2.9%)	0 (0.0%)
SCID	8 (5.7%)	4 (8.7%)
XIAP	4 (2.9%)	0 (0.0%)
Other immunodeficiencies^c^	13 (9.3%)	3 (6.5%)
Other conditions^d^	6 (4.3%)	3 (6.6%)
HSCT performed (*n*, %)	115 (82.1%)	34 (73.9%)

Abbreviations: ALL, acute lymphoblastic leukaemia; AML, acute myeloid leukaemia; BMF, bone marrow failure; CGD, chronic granulomatous disease; CID, combined immunodeficiency; HSCT, haematopoietic stem cell transplantation; IPEX, immune dysregulation, polyendocrinopathy, enteropathy, X‐linked; JMML, juvenile myelomonocytic leukaemia; MDS‐EB, myelodysplastic syndrome with excess of blasts; RCC, refractory cytopenia of childhood; r/r, relapsed/refractory; SAA, severe aplastic anaemia; SCID, severe combined immunodeficiency; XIAP, X‐linked inhibitor of apoptosis protein.

^a^ Inherited BMF include: Fanconi anaemia, dyskeratosis congenita, Diamond–Blackfan anaemia syndrome, congenital amegakaryocytic thrombocytopenia, infantile osteopetrosis, MDS with myelofibrosis and congenital thrombocytopenia.

^b^ CID comprises inborn errors of innate immunity that affect both B‐ and T‐cell function; when ‘CID’ was recorded as the official, non‐specific diagnosis for a patient, we retained it as a distinct entity, since this represented the most precise diagnosis available.

^c^ Other immunodeficiencies include various rare immunodeficiencies ≤ 2 cases: LRBA/CTLA‐4 deficiency, IPEX syndrome, Griscelli syndrome, leukocyte adhesion deficiency type 1, activated PI3KD syndrome, CD40L deficiency, CDC42‐associated neonatal myeloproliferation and autoinflammation, nemo‐like deficiency syndrome, X‐linked lymphoproliferative disease and STAT1‐/STAT3‐associated immunodeficiencies.

^d^ Other conditions include: essential thrombocythemia, hemophagocytic lymphohistiocytosis, mucopolysaccharidosis type 1, metachromatic leukodystrophy, sickle cell disease and thalassaemia.

* *p* < 0.05.

### 3.2 Reduction of the IFD Rate After Implementation of Environmental Preventive Measures

**FIGURE 2:**
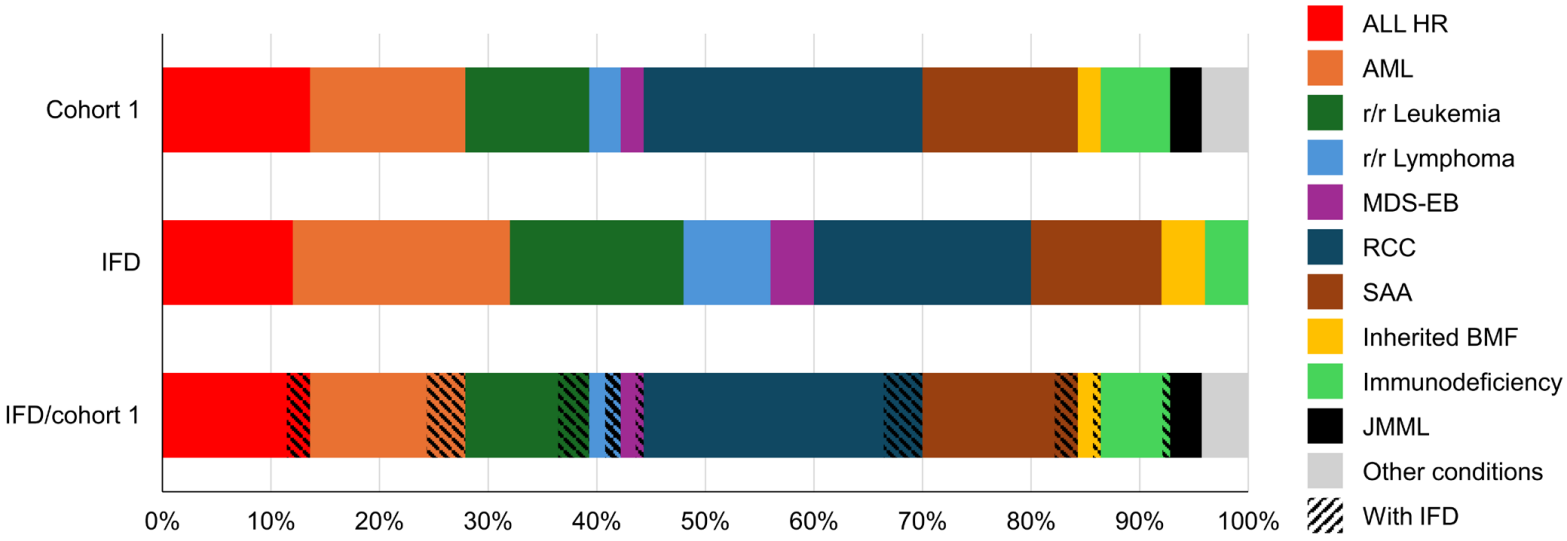
Underlying diagnosis in patients with IFD (*n* = 25) in Cohort 1 (*n* = 140). Stacked bar chart representing the disease distribution in Cohort 1 (upper bar), in the patients with IFD (central bar), and highlighting the portion of IFD cases per disease group (lower bar). Patients with IFD are displayed in striped baulks. Other conditions include essential thrombocythemia, haemophagocytic lymphohistiocytosis, mucopolysaccharidosis type 1, metachromatic leukodystrophy, sickle cell disease and thalassaemia. ALL, acute lymphoblastic leukaemia; AML, acute myeloid leukaemia; BMF, bone marrow failure; HSCT, haematopoietic stem cell transplantation; JMML, juvenile myelomonocytic leukaemia; MDS‐EB, myelodysplastic syndrome with excess of blasts; RCC, refractory cytopenia of childhood; r/r, relapsed/refractory; SAA, severe aplastic anaemia. Please refer to Table S5 for the list of patients with immunodeficiencies developing IFD.

During the study period, 25 IFD cases were identified (16.0% proven, 56.0% probable and 28.0% possible). Most IFD cases were pulmonary aspergillosis (76.0%), followed by invasive aspergillosis (20.0%) including one concurrent case of cutaneous aspergillosis and a single case of *Candida* sepsis (Table 3). In the four proven IFD, *Aspergillus fumigatus* was detected in three cases and *Candida kefyr* in one patient. Overall, IFD was predominantly detected in patients undergoing HSCT (Figure 2, Table 3, Figure S1 and Table S5). No seasonal variation of IFD incidence was observed.

No IFD occurred in Cohort 2 (0% vs. 17.9%, *p* = 0.002), that is, after implementation of environmental preventive measures (Table 3). This reduction was likely not attributable to the avoidance of fluconazole as antifungal prophylaxis in Cohort 2, since IFD rate and relative risks for IFD were comparable between patients treated with liposomal amphotericin B (LAB) and those receiving fluconazole in Cohort 1 (19.0% vs. 15.6%, *p* > 0.05; fluconazole RR = 0.9, 95% CI 0.4–3.2; LAB RR = 1.2, 95% CI 0.5–4.0). Additional analysis of Cohort 1 based on risk stratification according to local standards before 2021 (see Supporting Information ‘Local standards for prophylaxis of invasive fungal disease’) did not detect any difference in IFD rate or relative risk in patients treated with fluconazole (low‐risk: 10.5% vs. 7.4%, *p* > 0.05; fluconazole RR = 1.4, 95% CI 0.2–9.2; high‐risk: 20.0% vs. 21.2%, *p* > 0.05; fluconazole RR = 0.9, 95% CI 0.3–2.8).

### 3.3 Antifungal Prophylaxis and Therapy

Adherence to local standards for antifungal prophylaxis was extremely high in both cohorts (Cohort 1: 98.5%; Cohort 2: 100%, respectively; Table 2). Only two high‐risk patients in Cohort 1 did not receive antifungal prophylaxis: one infant with severe combined immunodeficiency (SCID) who underwent HSCT at the age of 3 months without conditioning, and one patient with high‐risk ALL who died of fulminant disease soon after diagnosis before prophylaxis could be initiated.

**TABLE 2:** Antifungal prophylaxis.

Antifungal prophylaxis	Cohort 1 (*n* = 140)	Cohort 2 (*n* = 46)	*p*
	*n* (%)	*n* (%)	
Liposomal amphotericin B	100 (71.5)	37 (80.4)	0.209
Fluconazole	32 (22.9)	0	< 0.001
Caspofungin	3 (2.1)	7 (15.2)	< 0.001
Voriconazole	3 (2.1)	0	0.312
Posaconazole	0	1 (2.2)	0.080
Isavuconazole	0	1 (2.2)	0.080
None	2 (1.4)	0	0.415

Abbreviation: *n*, number.

LAB represented the most commonly used prophylactic agent across both cohorts (71.5% and 76.2%). In Cohort 2, fluconazole administration was stopped in accordance with revised local standards, while caspofungin use increased (Table 2). Two patients received individualised antifungal prophylaxis: one boy with a third ALL relapse received oral posaconazole due to a prior anaphylactic reaction to amphotericin B and refusal of daily caspofungin infusion. Another patient with relapsed AML undergoing HSCT received caspofungin due to amphotericin B allergy. Following molecular relapse, the patient was later switched to isavuconazole during venetoclax/azacitidine therapy to reduce clinic visits and limit drug–drug interactions [23]. Seventeen patients required modifications to their prophylactic regimen (for details see Table S3).

Among 19 patients on prophylactic LAB, 10 underwent empirical or preemptive escalation to a therapeutic dosing (3 mg/kg/day) for persistent fever or galactomannan positivity in serum. All were subsequently transitioned to mould‐active azoles following either IFD‐typical chest CT findings (*n* = 7) or positivity of microbiological, molecular (PCR) or antigen testing in bronchoalveolar lavage (BAL) specimens (*n* = 3). Eight additional patients were switched directly from prophylactic LAB to mould‐active azoles after galactomannan positivity (*n* = 4) and/or IFD‐compatible CT findings (*n* = 5). One patient initially treated with caspofungin for suspected candidiasis was switched to voriconazole upon confirmation of aspergillosis.

Five patients developed IFD while on fluconazole prophylaxis. Of these, three received preemptive LAB following galactomannan positivity, one was treated with caspofungin after *Candida* spp. was detected in blood, and one was switched from caspofungin to LAB after developing mould‐typical pulmonary infiltrates. One previously published patient with immunodeficiency underwent surgical resection of pulmonary aspergillosis and remains alive without residual disease [19].

### 3.4 Outcome

Overall mortality was 13.6% (*n* = 19/140) in Cohort 1 and 6.5% (*n* = 3/46) in Cohort 2 (*p* = 0.19, Table 3). Progression or relapse of the underlying malignancy accounted for 26% and 33% of deaths, respectively. The leading causes of death were treatment‐related complications including multi‐organ failure, sepsis and uncontrolled viral infections, accounting collectively for approximately 65% of deaths across both cohorts. Although IFD was the primary cause of death in only 10% of deceased patients in Cohort 1, residual IFD was present in eight of nine patients who developed IFD and died. In contrast, among patients who experienced IFD and survived, 11 of 16 achieved complete fungal clearance, whereas five showed residual fungal disease. Overall, IFD and particularly residual IFD were associated with a significantly higher mortality (IFD: *p* < 0.0001, residual IFD: *p* < 0.000001; Table S6).

**TABLE 3:** Patient outcome and IFD characteristics.

Patient status	Cohort 1 (*n* = 140)	Cohort 2 (*n* = 46)
	*n* (%)	*n* (%)
Alive	121 (86.4)	43 (93.5)
Deceased	19 (13.6)	3 (6.5)
r/r cancer	5 (26.3)	1 (33.3)
Progressive IFD	2 (10.5)	0 (0.0)
Others incl. TRM	12 (63.2)	2 (66.7)
Invasive fungal disease according to EORTC/MSGERC		
Total	25 (17.9)*	0 (0.0)
Possible	7 (28.0)	—
Probable	14 (56.0)	—
Proven	4 (16.0)	—
Pulmonary aspergillosis	19 (76.0)	—
Invasive aspergillosis	5 (20.0)	—
Candida sepsis	1 (4.0)	—
Time point of IFD diagnosis		—
Prior to HSCT	9 (36.0)	—
During/after HSCT	13 (52.0)	—
No HSCT^a^	3 (12.0)	—
IFD status in patients alive	16 (64.0)	—
Restitutio ad integrum	11 (68.8)	—
Residual IFD^b^	5 (31.2)	—
IFD status in deceased patients	9 (36.0)	—
Restitutio ad integrum	1 (11.1)	—
Residual IFD	8 (88.9)	—

Abbreviations: HSCT, haematopoietic stem cell transplantation; IFD, invasive fungal disease; *n*, number; r/r, relapsed/refractory.

^a^ Three patients who developed IFD did not require HSCT: one AML and two high‐risk ALL.

^b^ Residual IFD was defined as microbiological, serological, or imaging evidence of IFD present at the last documented follow‐up contact with the treating centre. Restitutio ad integrum was defined as the absence of residual IFD.

* *p* < 0.01.

## 4 Discussion

This single‐centre retrospective study investigated the IFD incidence in two cohorts of paediatric high‐risk cancer and transplant patients in a large European childhood cancer centre under markedly different environmental preventive conditions.

Consistent with previous reports, intensified measures to reduce fungal exposure significantly lowered IFD incidence [17, 18, 24, 25, 26]. Indeed, no IFD was observed during the study period following relocation to the new building. The oncology branch of the former children's hospital was constructed in 1993 and further expanded in 2005. Since then, no major renovation was conducted (Table S2). Given that evidence from non‐randomised trials suggests that targeted measures such as removing carpets and replacing potentially colonised ceiling tiles may reduce IFD rate [27], the aging infrastructure of the former building may have contributed to the elevated IFD incidence in Cohort 1.

The new children's hospital provides strict structural separation for immunocompromised patients. In contrast, in the former hospital, patients at high risk for IFD occasionally transited through areas with air drafts and shared proximity to common passageways; under those conditions, airborne exposure to *Aspergillus* spores would be expected to be comparable to outdoor levels. However, in Cohort 1, IFD cases were homogeneously distributed throughout the year and did not correlate with the seasonal variation in outdoor spore concentrations, which are typically highest between June and September [28]. Although both cohorts included summer months, the difference in observation periods (56 vs. 12 months in Cohorts 1 and 2, respectively) and the resulting unequal exposure to high‐concentration periods (June to September) may have potentially inflated IFD rates in Cohort 1.

Although IFD incidence markedly decreased after relocation (17.9% vs. 0%), the rates in Cohort 1 exceeded those reported in previous multicentre studies (11% in Cesaro et al. and 6% in Lehrnbecher et al.) [1, 29]. This discrepancy likely reflects differences in cohort composition, as our analysis focused on high‐risk patients (haematological malignancies and HSCT) and excluded low‐risk situations like autologous HSCT [1, 22, 29, 30, 31]. Furthermore, possible IFD was included in the analysis of the primary endpoint to avoid underestimating the true infection burden given the elevated baseline risk of our cohort, whereas other studies included only probable and proven IFD. When analyses were restricted to probable and proven IFD, our IFD rate (13%) approached those of previous multicentre studies [1, 29]. Additional factors may also have contributed to the per se elevated IFD rate in Cohort 1, including differences in prophylaxis regimens and environmental conditions like construction activity in the vicinity of the former hospital.

Until 2021, fluconazole was considered an acceptable prophylactic option for high‐risk patients based on ECIL‐4 guidelines, resulting in substantial variability in prophylaxis regimens [32]. Cesaro et al. reported fluconazole prophylaxis use in 70% of patients who developed IFD, with *Candida* spp. accounting for approximately 50% of IFD cases, highlighting both rising fluconazole resistance and the need to adapt regimens to epidemiological changes [29, 33]. In our cohort, the only case of *Candida* sepsis occurred in a patient with relapsed anaplastic large‐cell lymphoma during a prolonged neutropenic phase while on fluconazole prophylaxis; the isolated *Candida kefyr* was subsequently confirmed fluconazole‐resistant. Additionally, several retrospective studies have reported lower IFD rates with mould‐active prophylaxis in patients with AML and high‐risk ALL compared to fluconazole [34, 35]. In line with these observations, ECIL‐8 now recommends mould‐active prophylaxis in high‐risk patients [6].

In our study, LAB was the primary antifungal prophylaxis in over 70% of patients. Despite frequent side effects (e.g., allergic reactions), its twice‐weekly dosing schedule and intravenous administration offer practical advantages during inpatient stays and the post‐HSCT period compared to other antifungals. Prophylaxis changes were occasionally required due to organ toxicity (e.g., nephrotoxicity), compliance (some adolescent patients preferred oral formulations where possible) or drug interaction profiles (Table S3).

While the shift towards echinocandins, LAB and mould‐active azoles represents a meaningful advance in antifungal prophylaxis, ongoing surveillance of fungal epidemiology and resistance patterns remains imperative to detect emerging resistant strains.

This study had several limitations inherent to its retrospective design; however, the planned hospital relocation offered a unique opportunity to assess the impact of environmental conditions while keeping other relevant variables largely unchanged—a comparison that would be ethically unacceptable in a randomised controlled trial given the precautionary principle and the vulnerability of highly immunocompromised patients (Article 191, EUR‐Lex) [36]. As the relocation was a recent event, Cohort 1 covered a substantially longer time period than Cohort 2 (56 vs. 12 months), resulting in significant differences in cohort size and follow up, possibly leading to selection bias. Nevertheless, from the experience of Cohort 1 we would not expect IFD to manifest at a later timepoint outside our study period in Cohort 2. The cohorts were also separated by a 4‐year interval, during which unrecorded minor changes in supportive care, graft‐versus‐host disease prophylaxis or chemotherapy intensity may have occurred, representing potentially unrecognised confounding factors. The total number of IFD cases was insufficient for comprehensive multivariate analysis accounting for additional potential risk factors (e.g., prolonged neutropenia, conditioning, GvHD, mucositis, underlying disease; see Table S4) and possible bias (e.g., effect of empirical treatment or change of prophylaxis). Nevertheless, the available sample size was sufficient to demonstrate a significant reduction of IFD incidence following environmental interventions, and to confirm the association between IFD and mortality, consistent with reports in critically ill and oncologic adult populations [37, 38, 39, 40].

## 5 Conclusion

This study demonstrated that comprehensive environmental preventive measures are able to significantly reduce IFD incidence in paediatric high‐risk cancer and transplant patients, even within a clinical setting already adhering to contemporary antifungal prophylaxis standards. These findings underscore the independent and additive value of structural and hygienic interventions alongside pharmacologic prophylaxis. Furthermore, IFD and particularly residual IFD were significantly associated with mortality, reinforcing the clinical imperative for surveillance, early diagnosis and effective treatment in high‐risk patients. Further research is needed to identify which patient subgroups benefit most from cost‐intensive environmental protective measures—particularly in resource‐limited settings—and to optimise antifungal prophylaxis in high‐risk cohorts.

## Author Contributions

**Markus Hufnagel:** writing – review and editing, conceptualization. **Tobias Feuchtinger:** writing – review and editing, resources. **Stefano Malvestiti:** writing – original draft, writing – review and editing, visualization, formal analysis, data curation, conceptualization, methodology. **Brigitte Strahm:** data curation, writing – review and editing, resources. **Alexander Puzik:** supervision, conceptualization, writing – original draft, writing – review and editing, data curation, visualization. **Carsten Speckmann:** writing – review and editing, data curation, resources. **Felicia Andresen:** data curation, writing – review and editing.

## Funding

The authors have nothing to report.

## Conflicts of Interest

The authors declare no conflicts of interest.

## Supporting information


**Figure S1:** Disease spectrum of patients with IFD.
**Table S1:** Definition of IFD according to Donnelly et al.
**Table S2:** Environmental conditions.
**Table S3:** Changes in antifungal prophylaxis for individual patients.
**Table S4:** Risk of IFD in the whole cohort (*n* = 186) according to subgroups.
**Table S5:** List of immunodeficiencies.
**Table S6:** Mortality rate according to IFD (*n* = 186).
**Table S7:** Antifungal agents and doses.

## Data Availability

Datasets on which the conclusions of the paper rely are stored according to local data security policy at the Medical Center of the University of Freiburg. The datasets generated and/or analysed during the current study are available from the corresponding author on reasonable request.
