# Supplementary material for: The Underestimated Role of Environmental Factors in the Prevention of Invasive Fungal Disease: Experience From a European Childhood Cancer Centre
Source: Mycoses. 2026 Jun 29;69(7):e70204. doi: 10.1111/myc.70204 (PMC13312149; doi:10.1111/myc.70204)
Supplement: Supplementary file 1 — Figure S1: Disease spectrum of patients with IFD. Table S1: Definition of IFD according to Donnelly et al. Table S2: Environmental conditions. Table S3: Changes in antifungal prophylaxis for individual patients. Table S4: Risk of IFD in the whole cohort (n = 186) according to subgroups. Table S5: List of immunodeficiencies. Table S6: Mortality rate according to IFD (n = 186). Table S7: Antifungal agents and doses. [file MYC-69-e70204-s001.docx]

**Local standards of procedures (SOP) for prophylaxis of invasive fungal disease**

Pharmacologic prevention of IFD was performed according to local SOPs. They define risk groups for IFD and provide recommendations for the selection of pharmacological prophylaxis. High-risk factors listed in the local guidelines are described in the main manuscript. Among patients with inborn errors of innate immunity, CGD is considered as high-risk. Other immunodeficiencies requiring HSCT and inherited bone marrow failure syndromes not associated with severe neutropenia were considered as non-high-risk. Until 2021, non-high-risk HSCT patients received non-mold-active agents (fluconazole) as antifungal prophylaxis. Overall, 33 patients in cohort 1 received fluconazole as prophylaxis (Supplementary Table S3). Among the 25 IFD cases recorded, only 5 were diagnosed in patients on a fluconazole prophylaxis. Based on national evidence suggesting an increased protective effect of mold-active agents compared to fluconazole, since 2021 all patients undergoing HSCT have primarily received mold-active agents, such as liposomal amphotericin B [12]. This represented the only modification of the local standards for IFD prevention during the study period. In cases of allergies, toxicities or rarely specific case-based rationales, the initial use of alternative antifungal agents was permitted. Supplementary Table S7 summarizes the antifungal agents administered, including their dosage, routes of administration and corresponding therapeutic drug monitoring (TDM). Antifungal prophylaxis was administered post-transplant until discontinuation of immunosuppressive therapy or until end of chemotherapy.

TABLE S1 Definition of IFD according to Donnelly, J.P., et al. [11]

| Proven IFD |
| --- |
| Microscopic analysis from sterile material (needle aspiration, biopsy) |
| - Direct microscopic examination of tissue with hyphae or melanized yeast-like forms + associated tissue damage |
| - Direct microscopic examination of tissue with yeast cell/ pseudohyphae or true hyphae |
| Culture |
| - Recovery of mold by culture from normally sterile or radiologically abnormal site (BAL, cranial sinus cavity, urine) |
| - Recovery of yeast in a sample by culture obtained from normally sterile site (e.g. drain placed <24hrs) |
| Blood |
| - Positive blood culture for mold |
| - Positive blood culture for yeast/yeast-like fungi |
| Tissue DNA by PCR |
| - Fungal DNA amplification and sequencing by PCR + microscopic evidence of molds in formalin-fixed paraffin-embedded tissue |
| - Fungal DNA amplification and sequencing by PCR + microscopic evidence of yeast in formalin-fixed paraffin-embedded tissue |
| Probable IFD  (= presence of ≥ 1 host factor + ≥ 1 clinical criterion + ≥ 1 mycological criterion) |
| Host factors |
| - Prolonged neutropenia (<500/µL for >10 days), temporally related to onset of IFD |
| - Hematologic malignancy |
| - Allogeneic HSCT |
| - Solid organ transplant |
| - Prolonged corticosteroid therapy (≥ 0.3mg/kg/d ≥ 3 weeks in past 60 days) |
| - Treatment with B-cell-immunosuppressants (TKIs, e.g. ibrutinib, etc.) |
| - Treatment with T-cell immunosuppressant (cyclosporine A, TNF-alpha blockers, monoclonal antibodies (alemtuzumab), nucleoside analogs in past 90 days |
| - Inherited severe immunodeficiency |
| - Acute GvHD grade III and IV involving gut, lungs, or liver; refractory to first-line treatment with steroids |
| Clinical criteria |
| - CT shows 1 of 4 signs: dense, well circumscribed lesions w/ or w/o halo sign, air-crescent sign, cavity, wedge-shaped and segmental or lobar consolidation |
| - Tracheobronchial ulceration, nodule, pseudomembrane, plaque, eschar on bronchoscopy |
| - Imaging of sinusitis + 1 of 3 signs: acute localized pain, nasal ulcer w/ black eschar, extension from paranasal sinus across bone barriers |
| - CNS: 1 of 2 signs: focal lesions on imaging, meningeal enhancement on MRI/CT |
| - 1 of 2 signs after candidemia within previous 2 weeks: small, target-like abscesses in liver/spleen, progressive retinal exudates |
| Mycological criteria |
| - Direct test: mold in sputum, BAL, bronchial brush, sinus aspirate: presence of fungal elements, recovery by culture of mold |
| - Galactomannan positive in plasma or serum (≥ 1.0) for aspergillosis |
| - Galactomannan positive in BAL (≥ 1.0) |
| - Galactomannan positive in CSF (1.0) or serum/plasma ≥ 0.7 + BAL ≥ 0.8 for aspergillosis |
| - Galactomannan positive in serum/plasma ≥ 0.7 and BAL ≥ 0.8 for aspergillosis |
| - Aspergillus PCR: positive in plasma, serum, whole blood in ≥ 2 consecutive tests |
| - Aspergillus PCR: BAL fluid positive in ≥ 2 duplicates |
| - Aspergillus PCR: 1 PCR test positive in plasma/serum/whole blood and 1 PCR positive in BAL fluid |
| - Aspergillus species recovered by culture from sputum, BAL, bronchial brush, aspirate |

TABLE S1 (continued)

| Possible IFD  (= presence of ≥ 1 host factor + ≥ 1 clinical criterion, without mycological criteria) |
| --- |
|  |
| Probable Candidiasis  (= presence of ≥ 1 host factor + ≥ 1 clinical criterion + ≥ 1 mycological criterion) |
| Host factors |
| - Prolonged neutropenia (<500/µL for >10 days), temporally related to onset of IFD |
| - Hematological malignancy |
| - Allogeneic HSCT |
| - Solid organ transplant |
| - Prolonged corticosteroid therapy (≥ 0.3mg/kg/d > 3 weeks in past 60 days) |
| - Treatment with B-cell-immunosuppressants (TKIs, e.g. ibrutinib, etc.) |
| - Treatment with T-cell immunosuppressant (cyclosporine A, TNF-alpha blockers, monoclonal antibodies (alemtuzumab), nucleoside analogs in past 90 days |
| - Inherited severe immunodeficiency |
| - Acute GvHD grade III and IV involving gut, lungs, or liver; refractory to first-line treatment with steroids |
| Clinical features (≥ 1 of 2 entities after candidemia within previous 2 weeks) |
| - Small, target-like abscesses in liver or spleen (bull's-eye-lesions) or in brain or meningeal enhancement |
| - Progressive retinal exudates or vitreal opacities on ophthalmologic examination |
| Mycological criteria |
| - Beta-D-Glucan (Fungitell®) ≥ 80 ng/L (pg/mL) detected in ≥ 2 consecutive serum samples |
| - Positive T2Candida Panel®^#^ |
| # T2Candida Panel® (T2 Biosystems, Inc., MA) is a molecular diagnostic assay approved by the US Food and Drug Administration for the detection of selected *Candida spp.* (*C. albicans, C. tropicalis, C. parapsilosis, C. krusei, and C. glabrata*) from whole blood specimens.  Abbreviations: BAL, bronchoalveolar lavage; CSF, cerebrospinal fluid; CT, computed tomography; GVHD, graft-versus-host disease HSCT, hematopoietic stem cell transplant; IFD, invasive fungal disease; MRI, magnetic resonance imaging; PCR, polymerase chain reaction; TKI, tyrosine kinase inhibitor; TNF, tumor-necrosis-factor w/, with; w/o, without. |

TABLE S2 Environmental conditions

| Criteria | Conditions | |
| --- | --- | --- |
|  | Former children’s hospital  (cohort 1) | New children’s hospital  (cohort 2) |
| Years of  construction | Main building: 1956  Oncology branch: 1991 – 1993  Extension of oncology branch: 2003 – 2005 | 2021 – 2024 |
| Localization | RG 1-2: patient rooms were partially in a separate unit. Occasionally, patients were accommodated in units shared with other specialties; sometimes rooms were located in close proximity to passageways. | RG 1-3: patient rooms are in separate structural unit and distant from passageways to other departments. |
|  | RG 3: patient rooms are in separate structural unit and distant from passageways to other departments. |  |
| Room | RG 1-2: single, double or triple rooms | RG 1-2: single or double rooms |
|  | RG 3: single-room, sealed-room, adequate size to host, positive pressure. | RG 3: single-room, sealed-room, adequate size to host, positive pressure. |
| Sanitary | RG 1-2: Floor sanitary facility shared by multiple patients. | RG 1-2: En-suite sanitary facility every two patients. |
|  | RG 3: En-suite sanitary facility for each patient with splash guard. | RG 3: En-suite sanitary facility for each patient with splash guard. |
| Anteroom | Patient´s room is separated from the rest of the ward by an anteroom with two doors and positive pressure. | Patient´s room is separated from the rest of the ward by an anteroom with two doors and positive pressure. |
| Additional  separation | Sealed windows in 5 single rooms dedicated to allogeneic HSCT | Sealed windows in the whole HSCT area;  Additionally, two-door physical barrier between the aisle of the HSCT area and the remainder of the oncology ward. |
| Filtration | High-efficiency particulate air within 5 single-rooms and corresponding anterooms. | High-efficiency particulate air within 8 single-rooms and corresponding anterooms, as well as in the aisle within the HSCT area. |
| Air direction | The air flow is directed from the patient to exhaust vents. | The air flow is directed from the patient to exhaust vents. |
| Monitoring /  Surveillance | HEPA filter exchange and monitoring of leak-tight air filter every 2 – 3 years  Air-pressure surveillance | HEPA filter exchange and monitoring of leak-tight air filter every 1 – 2 years  Air-pressure surveillance  Air-flow direction surveillance |
| Abbreviations: RG, KRINKO risk group; HSCT, hematopoietic stem cell transplantation. | | |

TABLE S3 Changes in antifungal prophylaxis for individual patients

| Cohort 1 (n=140) |  | |  |  |
| --- | --- | --- | --- | --- |
|  | Initial antifungal prophylaxis | | Final antifungal prophylaxis | Description |
|  | n (%) | | n (%) |  |
| Liposomal amphotericin B | 97 (70.3) | | 100 (71.5) | 1xPt. switched from LAB to VOR due to compliance; 3xPt. switched from ITR to LAB due to interaction profile; 1xPt. switched from MIC to LAB due to local standard. |
| Fluconazole | 33 (23.9) | | 32 (22.9) | 1xPt. switched from FLU to CAS due to interaction profile |
| Caspofungin | 2 (1.4) | | 3 (2.1) | 1xPt. switched from FLU to CAS due to interaction profile |
| Voriconazole | 2 (1.4) | | 3 (2.1) | 1xPt. switched from LAB to VOR due to compliance |
| Itraconazole | 3 (2.2) | | 0 (0.0) | All Pt. switched from ITR to LAB due to interaction profile |
| Micafungin | 1 (0.7) | | 0 (0.0) | Pt. was switched to LAB due to local standard. |
| None | 2 (1.4) | | 2 (1.4) | 1xPt. with SCID was transplanted without conditioning and 1xPt. died of fulminant disease before antifungal prophylaxis could be started |
| Cohort 2 (n=46) |  | |  |  |
| Liposomal amphotericin B | 44 (95.8) | | 37 (80.4) | 7xPt. switched from LAB to CAS due to allergic reaction to LAB, 2xPt. switched to CAS due to AKI |
| Posaconazole | 1 (2.2) | | 1 (2.2) | 1x Pt. directly received POS after Dx of rALL due to PMHx positive for allergic reaction to LAB |
| Caspofungin | 1 (2.2) | | 7 (15.2) | 1xPt. received caspofungin at Dx of rAML due to PMHx positive for allergic reaction to LAB |
| Isavuconazole | 0 (0.0) | | 1 (2.2) | 1xPt. switched to ISA after engraftment due to compliance |
|  | | | | |
| Reasons for changing prophylaxis (n = 17) | | | |  |
|  | | n (%) | |  |
| Allergic reaction | | 7 (41.2) | |  |
| Non-conformity^#^ | | 4 (23.5) | |  |
| Compliance | | 2 (11.8) | |  |
| Interaction profile | | 2 (11.8) | |  |
| N/A | | 2 (11.8) | |  |
| # “Non-conformity” refers to patients who received antifungal prophylaxis before transfer to our medical center and whose prophylaxis at the referring hospital did not align with our standards. Prophylaxis was therefore changed following admission.  Abbreviations: AKI, acute kidney injury; CAS, caspofungin; Dx, diagnosis; FLU, fluconazole; ISA, isavuconazole; ITR, itraconazole; LAB, liposomal amphotericin B; MIC, micafungin; PMHx, past medical history; POS, posaconazole; Pt., patient; rALL, relapsed ALL; rAML, relapsed AML; SCID, severe combined immunodeficiency; VOR, voriconazole, N/A not available. | | | | |

TABLE S4 Risk of IFD in the whole cohort (n=186) according to subgroups

| Subgroup | IFD | no IFD | RR (95%CI) |
| --- | --- | --- | --- |
| Malignancy | 15 | 70 | 1.8 (0.8 - 3.8) |
| Immunodeficiency | 5 | 42 | 0.75 (0.3 - 1.9) |
| Inherited bone marrow failure syndrome | 5 | 40 | 0.8 (0.3 - 1.9) |
| Other conditions* | 0 | 9 | N/A |
| *Other conditions include: essential thrombocythemia, hemophagocytic lymphohistiocytosis, mucopolysaccharidosis type 1, metachromatic leukodystrophy, and sickle cell disease, thalassemia.  Abbreviations: HSCT, hematopoietic stem cell transplantation; IFD, invasive fungal disease; RR, relative risk; N/A, not available. | | | |

TABLE S5 List of immunodeficiencies

| Immunodeficiencies (n = 35) | n (%) | IFD (n) |
| --- | --- | --- |
| CGD | 7 (20.0) | 2 |
| CID* | 4 (11.4) | 0 |
| CTLA-4-deficiency | 2 (5.7) | 0 |
| Griscelli syndrome | 2 (5.7) | 1 |
| IPEX syndrome | 2 (5.7) | 0 |
| LRBA | 2 (5.7) | 0 |
| SCID | 7 (20.0) | 0 |
| XIAP | 4 (11.4) | 1 |
| Others | 5 (14.3) | 1 |
| “Others” include various rare immunodeficiencies < 2 cases.  * CID comprises inborn errors of innate immunity that affect both B‑ and T‑cell function; when “CID” was recorded as the official, non‑specific diagnosis for a patient, we retained it as a distinct entity in the table, since this represented the most precise diagnosis available.  Abbreviations: CGD, chronic granulomatous disease; CID, combined immunodeficiency; CTLA-4, cytotoxic t-lymphocyte-associated protein 4; IFD, invasive fungal disease; IPEX, immune dysregulation, polyendocrinopathy, enteropathy, X-linked; LRBA, lipopolysaccharide- responsive and beige-like anchor protein; SCID, severe combined immunodeficiency; XIAP, X-linked inhibitor of apoptosis protein. | | |

TABLE S6 Mortality rate according to IFD (n=186)

|  |  | Alive (n, %) | Deceased (n, %) | OR (95%CI) |
| --- | --- | --- | --- | --- |
| IFD | + | 16 (8.6) | 9 (4.8) | 6.40 (2.37 – 17.31) |
|  | – | 148 (79.6) | 13 (7.0) |  |
| Residual IFD* | + | 5 (2.7) | 8 (4.3) | 18.17 (5.24 – 63.03) |
|  | – | 159 (85.5) | 14 (7.5) |  |
| Abbreviations: IFD, invasive fungal disease; OR, odds ratio; –, absence; +, presence.  *Residual IFD was defined as microbiological, serological, or imaging evidence of IFD present at the last documented follow-up contact with the treating center. | | | | |

TABLE S7 Antifungal agents and doses

| Antifungal agent | Dosage and administration | TDM |
| --- | --- | --- |
| Liposomal Amphotericin B | 2.5 mg/kgBW/d, 2x/wk, i.v. | No TDM required |
| Fluconazole | 100 mg/m^2^BS/d, qd, i.v. / p.o. | No TDM required |
| Caspofungin | Day 1: 70 mg/m^2^BS, i.v.  On following day: 50 mg/m^2^BS/d, qd, i.v. | No TDM required |
| Voriconazole | Age-adjusted and BW-adjusted dosage and administration:  Age 2 – 4 yo and BW < 50kg:  i.v.:  Day 1: 9 mg/kg/SD, bid  Following days: 8 mg/kg/SD, bid  p.o.:  9 mg/kg/SD, bid  If BW ≥ 40 kg – Day 1: 400 mg/SD, bid  Following days: 200mg/SD, bid  Age ≥ 15 yo, or age > 12 yo and BW > 50 kg:  i.v.:  Day 1: 6 mg/kg/SD, bid  Following days: 4 mg/kg/SD, bid  p.o.:  Day 1: 400 mg/SD, bid  Following days: 200 mg/SD, bid | 1 – 6 mg/L |
| Posaconazole | Age ≥ 2 y/o: age-adjusted and BW-adjusted dosage and administration according to producer recommendation. | 0.7 – 4 mg/L |
| Itraconazole | Age ≥ 2 yo:  2.5 mg/kgBW/SD, bid, both i.v. / p.o. | 0.7 – 4 mg/L |
| Isavuconazole | Age ≥ 1 y/o: age-adjusted and BW-adjusted dosage and administration according to producer recommendation. | Trough level < 5 mg/L |
| Abbreviations: BW, body weight; BS; body surface; d, day; i.v., intravenous, p.o., per os; SD, single dose; TDM, therapeutic drug monitoring; wk, week; yo, years old. | | |

|  |  | n | % (IFD) | % (Disease group) |
| --- | --- | --- | --- | --- |
|  | ALL HR | 3 | 12.0 | 15.8 |
|  | AML | 5 | 20.0 | 25.0 |
|  | r/r Leukemia | 4 | 16.0 | 25.0 |
|  | r/r Lymphoma | 2 | 8.0 | 50.0 |
|  | MDS-EB | 1 | 4.0 | 33.3 |
|  | Immunodeficiency | 5 | 20.0 | 13.9 |
|  | RCC | 3 | 12.0 | 15.0 |
|  | SAA | 1 | 4.0 | 33.3 |
|  | Inherited BMF | 1 | 4.0 | 11.1 |

FIGURE S1 Disease spectrum of patients with IFD. The table under the pie chart shows the absolute number of IFD per disease group, and their percentages calculated relative to the number of IFD (central column; n = 25), or to the number of each disease group in cohort 1 (right column; ALL HR = 3 /19, AML 5/20, r/r Leukemia = 2/16, r/r Lymphoma = 2/4, MDS-EB = 1/3, Immunodeficiency = 5/36, RCC = 3/20, SAA = 1/3, Inherited BMF = 1/9). Abbreviations: ALL, acute lymphoblastic leukemia; AML, acute myeloid leukemia; BMF, bone marrow failure; HSCT, hematopoietic stem cell transplantation; JMML, juvenile myelomonocytic leukemia; MDS-EB, myelodysplastic syndrome with excess of blasts; RCC, refractory cytopenia of childhood; r/r, relapsed / refractory; SAA, severe aplastic anemia. Please refer to Supplemental Table S5 for the list of immunodeficiencies developing IFD.
